# Cardiopulmonary Exercise Testing Correlates with Quantitative Left Ventricular [^99m^Tc]-DPD Uptake in Transthyretin Amyloid Cardiomyopathy

**DOI:** 10.3390/jcm14092999

**Published:** 2025-04-26

**Authors:** Nikita Ermolaev, René Rettl, Robin Willixhofer, Christina Kronberger, Michael Poledniczek, Lena Marie Schmid, Franz Duca, Christina Binder, Mahshid Eslami, Dietrich Beitzke, Christian Loewe, Marcus Hacker, Andreas Kammerlander, Johannes Kastner, Jutta Bergler-Klein, Raffaella Calabretta, Roza Badr Eslam

**Affiliations:** 1 Division of Cardiology, Department of Internal Medicine II, Medical University of Vienna, 1090 Vienna, Austria; nikita.ermolaev@meduniwien.ac.at (N.E.); rene.rettl@meduniwien.ac.at (R.R.); robin.willixhofer@meduniwien.ac.at (R.W.); christina.kronberger@meduniwien.ac.at (C.K.); lena.schmid@meduniwien.ac.at (L.M.S.); franz.duca@meduniwien.ac.at (F.D.); christina.binder@meduniwien.ac.at (C.B.); mahshid.eslami@meduniwien.ac.at (M.E.); andreas.kammerlander@meduniwien.ac.at (A.K.); johannes.kastner@meduniwien.ac.at (J.K.); jutta.bergler-klein@meduniwien.ac.at (J.B.-K.); 2 Division of Cardiovascular and Interventional Radiology, Department of Biomedical Imaging and Image-Guided-Therapy, Medical University of Vienna, 1090 Vienna, Austria; dietrich.beitzke@meduniwien.ac.at (D.B.); christian.loewe@meduniwien.ac.at (C.L.); 3 Division of Nuclear Medicine, Department of Biomedical Imaging and Image-Guided Therapy, Medical University of Vienna, 1090 Vienna, Austria; marcus.hacker@meduniwien.ac.at (M.H.); raffaella.calabretta@meduniwien.ac.at (R.C.)

**Keywords:** cardiac amyloidosis, transthyretin amyloidosis, heart failure, cardiopulmonary exercise testing, DPD quantification

## Abstract

**Background/Objectives**: Patients with transthyretin amyloid cardiomyopathy (ATTR-CM) often experience significantly reduced functional capacity due to myocardial involvement. Cardiopulmonary exercise testing (CPET) is the gold standard to quantify functional capacity, and ^99m^Tc-DPD scintigraphy and SPECT/CT have proven to be highly effective tools for diagnostic and disease monitoring. We aimed to investigate the complementary role and correlation between both methods, focusing on their combined potential as a strong prognostic framework for monitoring disease progression and evaluating treatment efficacy. **Methods**: A total of 44 patients with diagnosed ATTR-CM, who underwent ^99m^Tc-DPD scintigraphy and SPECT/CT imaging as well as CPET, were included. All patients were divided into two groups based on the median DPD retention index (low DPD uptake: ≤5.0, n = 22; high DPD uptake: >5.0, n = 22). **Results**: The mean age was 78 years, with 82% of participants being male. Significant correlations were observed between peak VO_2_ and DPD retention index (r = −0.355, *p* = 0.018) as well as between peak VO_2_ at anaerobic threshold with DPD retention index (r = −0.391, *p* = 0.009). Interestingly, there was no strong correlation between VE/VCO_2_ slope and the retention index. A strong association was identified between cardiac biomarkers and peak VO_2_, specifically for NT-proBNP (r = −0.530, *p* < 0.001) and Troponin T (r = −0.431, *p* < 0.001). **Conclusions**: In ATTR-CM, significant correlations were observed between key CPET parameters and quantitative cardiac DPD uptake, which further reflects on disease severity and functional impairment. Our findings highlight the utility of integrating CPET and SPECT/CT for comprehensive patient assessment in ATTR-CM.

## 1. Introduction

Transthyretin amyloid cardiomyopathy (ATTR-CM) is a progressive condition characterized by the extracellular deposition of misfolded transthyretin proteins, leading to myocardial dysfunction [1]. These deposits disrupt the myocardial architecture, causing diastolic dysfunction, reduced cardiac output, and severe heart failure (HF) [2]. ATTR-CM is often an underdiagnosed cause of HF, with a recent study estimating its prevalence to be up to 25% in heterogeneous population cohorts [3]. Diagnostic delays are still common, given the overlap of symptoms with other cardiac conditions and the limited awareness of the disease. Early diagnosis of ATTR-CM is crucial, as timely interventions can mitigate disease progression and improve patient outcomes [4]. Advances in bone scintigraphy and non-invasive imaging have improved detection rates, yet functional evaluation through cardiopulmonary exercise testing (CPET) remains underutilized in this context.

Quantitative single-photon emission computed tomography (SPECT/CT) using tracers [^99m^Tc]-3,3-diphosphono-1,2-propanodicarboxylic acid (DPD) or [^99m^Tc]-pyrophosphate (PYP) has emerged as the gold standard in the diagnosis of ATTR-CM. These methods enable the visualization and quantification of amyloid deposits [5,6], with high amyloid burden correlating with worse outcomes [7]. However, patients diseased with ATTR-CM are experiencing a progressive shortness of breath and reduced functional capacity (FC), and imaging alone may not fully capture the functional implications of amyloid burden. While DPD scintigraphy provides a robust method for visualizing and quantifying amyloid deposits, it alone may not fully capture the functional implications of amyloid burden on the patient’s daily activities and exercise capacity. To bridge this gap, CPET should be used as a complementary tool.

CPET has been shown to be valuable in the assessment of patients with ATTR-CM [8,9,10]. Among evaluated parameters, peak oxygen consumption (VO_2_) and the ventilation to carbon dioxide production (VE/VCO_2_) slope have been shown to improve the evaluation of disease severity and functional impairment in patients with cardiac amyloidosis [8,11,12]. Based on the findings of Hwang et al. [13], cardiovascular magnetic resonance (CMR) and CPET together serve as a strong complementary predictor of clinical outcomes in patients with hypertrophic cardiomyopathy. However, no studies have comprehensively examined the correlation and role of CPET and quantitative DPD SPECT/CT, especially in patients with ATTR-CM.

Thus, this study aimed to explore the relationship between structural imaging and functional assessment by investigating the correlation between CPET-derived parameters and quantitative DPD SPECT/CT imaging and how these two diagnostic approaches affect patient outcome.

## 2. Materials and Methods

### 2.1. Setting and Study Design

The present study was conducted in the frame of a clinical registry (EC #1918/2019 and 1079/2023) at the Department of Internal Medicine II, Division of Cardiology at the Medical University of Vienna. This study was approved by the Ethics Committee of the Medical University of Vienna and conducted according to good clinical practice as outlined in the Declaration of Helsinki. All study participants gave written informed consent prior to enrolment.

### 2.2. Study Population

Patients with a confirmed diagnosis of ATTR-CM based on validated criteria between 2019 and 2023 at our specialized amyloidosis outpatient clinic were invited to participate in a prospective, clinical follow-up program. The program included regular clinical evaluations and systematic assessments of cardiac function. Eligibility for inclusion required participants (i) to be able to undergo CPET and [^99m^Tc]-DPD bone scintigraphy with additional quantitative thoracic SPECT/CT imaging; (ii) Perugini grade 2 or 3 cardiac uptakes on [^99m^Tc]-DPD bone scintigraphy in the absence of an abnormal serum free light chain ratio and monoclonal immunoglobulin in the serum and urine by immunofixation; and (iii) absence of disease-specific therapy prior to CPET assessment and quantitative [^99m^Tc]-DPD SPECT/CT imaging.

### 2.3. Diagnosis of Cardiac Transthyretin Amyloidosis

The diagnosis of ATTR-CM was determined based on the presence of HF symptoms combined with imaging findings consistent with ATTR-CM. Planar DPD bone scintigraphy was performed in accordance with the proposed recommendations previously reported by Gillmore et al. [5]. Aside from bone scintigraphy, all patients underwent mandatory screening for paraprotein and monoclonal protein, including the following laboratory tests: serum free light chain assay, as well as serum and urine immunofixation. Gene sequencing was performed if patients had given written consent for genetic analysis.

### 2.4. Cardiopulmonary Exercise Testing

CPET was conducted using individualized ramp protocols tailored to each patient’s functional status based on subjective daily physical activity and results of the 6MWT at baseline. Data acquisition included breath-by-breath gas analysis (Dual-Monitor Vyntus CPX SN 42600071, Carl Reiner, Vienna, Austria) and continuous ECG and heart monitoring (GE CAM USB CardioSoft 12-channel-PC-ECG, GE Healthcare, Wauwatosa, WI, USA), based on protocols previously described by Willixhofer et al. [8].

All parameters were assessed for up to two minutes during rest, during exercise, and up to 3 minutes during recovery. Analyzed CPET variables included peak VO_2_, VO_2_ at anaerobic threshold (AT), peak pulmonary ventilation (VE), peak oxygen pulse (peak O_2_ pulse), as well as VE/VCO_2_ slope. Peak VO_2_ was defined as the highest 30-s value reached and identified by the disproportionate rise in VE relative to VO_2_. The AT was determined using the V-slope method and validated by ventilatory equivalent and end-tidal methods [14]. Peak O_2_ pulse was determined by dividing VO_2_ (mL/min) by heart rate (beats/min). The VE/VCO_2_ slope was calculated as the slope of the linear relationship between VE and carbon dioxide production (VCO_2_) from the beginning of loaded exercise to the end of the isocapnic buffering period [15]. Peak VE was calculated by multiplying the respiratory rate by the volume of air exhaled during each breathing cycle (tidal volume).

### 2.5. Imaging Assessments

#### 2.5.1. DPD Scintigraphy and SPECT/CT Imaging

Nuclear imaging was conducted at the Division of Nuclear Medicine, Medical University of Vienna, using a Symbia Intevo SPECT/CT system (Siemens Medical Solutions AG, Erlangen, Germany). Planar whole-body scintigraphy was performed 2.5 h after intravenous [^99m^Tc]-DPD injection (720.0 MBq), followed by thoracic SPECT/CT imaging at 3.0 h. Imaging used a 180° configuration, 64 views (20 s/view), a 256 × 256 matrix, and a 15% energy window around the 142 keV photopeak. Low-dose CT was acquired for attenuation correction. Reconstruction used xSPECT/CT QUANT technology to standardize uptake values [16]. Planar images were visually graded per the Perugini classification [17], and left ventricle (LV) [^99m^Tc]-DPD uptake on SPECT/CT images was quantified using specialized software (Hermes Hybrid 3D software, Version 2.0, Hermes Medical Solutions, Stockholm, Sweden) on SPECT/CT images. As previously reported, the [^99m^Tc]-DPD retention index was calculated by the ratio of the standardized uptake value (SUV) of peak cardiac uptake to the SUV of peak vertebral uptake, multiplied by the SUV of peak paraspinal muscle uptake [18]. This index provides a normalized measure of amyloid deposition in the heart relative to other tissues, enhancing the specificity of [^99m^Tc]-DPD SPECT/CT imaging for quantifying cardiac LV involvement in amyloidosis.

#### 2.5.2. Cardiac Magnetic Resonance Imaging

Patients underwent cardiac magnetic resonance (CMR) imaging on a 1.5 Tesla scanner (Avanto Fit, Siemens Healthcare GmbH, Erlangen, Germany) according to standard protocols [19]. This included late gadolinium enhancement imaging using gadobutrol (Gadovist, Bayer Vital GmbH, Leverkusen, Germany) and T1-mapping to determine the extracellular volume (ECV).

#### 2.5.3. Transthoracic Echocardiography

Transthoracic echocardiography (TTE) was conducted by certified professionals using state-of-the-art equipment (GE Vivid E95, Vivid E9, and Vivid 7, GE Healthcare, Wauwatosa, WI, USA) following current guidelines [20]. Image interpretation was carried out after the assessment on a contemporary offline clinical workstation equipped with specialized software Version 204 (EchoPAC, GE Healthcare, Wauwatosa, WI, USA) by certified cardiologists.

### 2.6. Outcome Measures

A systematic search of the Austrian electronic health record system was conducted to assess the vital status and occurrence of heart failure-related hospitalizations among all 44 patients. These data were collected to support the outcome analysis, with the primary outcome defined as a composite of heart failure hospitalization or all-cause death.

### 2.7. Statistical Analysis

The analyses were performed using SPSS version 29.0 (IBM Corp, New York, NY, USA). Continuous variables were calculated as median and interquartile range (IQR), and categorical variables were reported as numbers and percentages. Comparisons of differences between variables among the two groups were made by the Chi-square test for categorical variables or the Mann–Whitney U test for continuous variables. Spearman correlation coefficients were used for correlation analysis. Survival was analyzed using Cox proportional hazards regression, with results presented as hazard ratios (HR) and corresponding Kaplan–Meier survival curves. Separate univariate and multivariate Cox regression models were used to evaluate the impact of key clinical, CPET, and imaging variables on the composite outcome endpoint. Kaplan–Meier estimate cut-off values for peak VO_2_ and VE/VCO_2_ slope were set at 14 mL/kg/min and 40, respectively, as previously reported by Badr Eslam et al. [9]. A significance level of *p* ≤ 0.05 was adopted for all hypothesis tests.

## 3. Results

### 3.1. Study Participants and Baseline Characteristics

**Figure 1:**
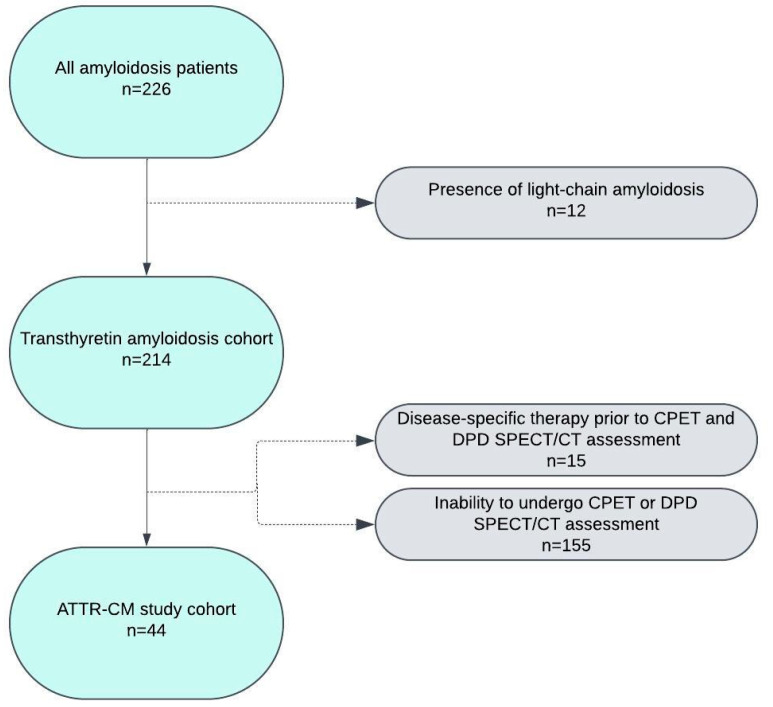
Total amyloidosis cohort and reasons for study exclusion. Abbreviations: CPET, cardiopulmonary exercise testing; DPD, [^99m^Tc]-3,3-diphosphono-1,2-propanodicarboxylic acid; SPECT/CT, Single-photon emission computed tomography. Figure was created with lucid.app.

The initial cohort consisted of 226 patients diagnosed with cardiac amyloidosis. Further detailed assessment of the cohort based on eligibility for this study led to a substantial reduction in the cohort size. A detailed flow of patient inclusion and exclusion throughout this study is depicted in Figure 1. The final study cohort included 44 patients with ATTR-CM, of whom 82% were male, with a median age of 78 (73.0–78.0) years. TTR gene sequencing identified 5 patients (11%) with a variant form (ATTRv-CM). In our cohort, the following mutations were observed: His108Arg (n = 4), Thr69Ile (n = 1). Study participants were divided into two cohorts based on the median [^99m^Tc]-DPD retention index– low uptake cohort (≤5.0, n = 22) and high uptake cohort (>5.0, n = 22).

Baseline characteristics were assessed for the overall group and the two subgroups (Table 1). Key demographic data such as age, sex, and mean body mass index (BMI) showed no significant differences between the groups. Participants demonstrated varying degrees of functional impairment, as reflected in the distribution across New York Heart Association (NYHA) classes II and III, with 50 and 36%, respectively. The median distance covered in the 6 min walk test (6MWT) was 422.0 m (IQR: 366.1–458.3). TTE demonstrated a highly thickened interventricular septum (18.0 mm, IQR: 17.4–20.2) and a preserved ejection fraction (51.0%; IQR: 47.1–53.0). Left ventricular dimensions remained stable, with an end-diastolic diameter of 43.0 mm (IQR: 42.0–45.5). However, left ventricular global longitudinal strain was notably reduced (12.0%; IQR: 13.5–11.4), which indicates a myocardial dysfunction.

The prevalence of coronary artery disease was significant between the low and high DPD uptake groups (45.0% vs. 14.0%, *p* = 0.045). Cardiac biomarker analysis showed a notable difference in NT-proBNP level (1364.0 vs. 2341.0 pg/mL, *p* = 0.029). CPET results indicated impaired functional capacity, with a median peak VO_2_ of 16.1 in the low uptake group and 12.7 mL/min/kg in the high uptake group, and VE/VCO_2_ slope of 39.2 and 39.7, respectively. Furthermore, peak workload was more impaired in the high uptake group with only 56 Watts, compared to the low uptake cohort, which reached 90 Watts (*p* = 0.003).

### 3.2. Correlation Between CPET Variables and Quantitative LV [^99m^Tc]-DPD Uptake

**Figure 2:**
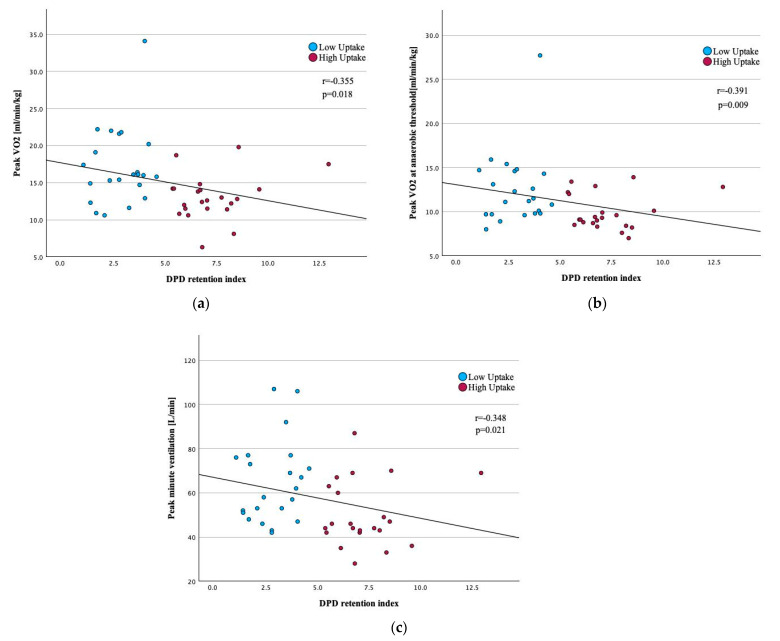
(**a**) Correlation between peak oxygen uptake and left ventricular [^99m^Tc]-DPD retention index; (**b**) Correlation between peak oxygen uptake at anaerobic threshold and left ventricular [^99m^Tc]-DPD retention index. (**c**) Correlation between peak minute ventilation and left ventricular [^99m^Tc]-DPD retention index. Abbreviations: [^99m^Tc]-DPD, [ ^99m^Tc]-3,3-diphosphono-1,2-propanodicarboxylic acid; VO_2_, oxygen consumption.

The correlation between CPET variables and the [^99m^Tc]-DPD retention index revealed a significant relationship, which indicates the impact of amyloid burden on functional capacity in patients with ATTR-CM. Peak VO_2_ demonstrated a moderate negative correlation with [^99m^Tc]-DPD retention index (r = −0.355, *p* = 0.018), as illustrated in Figure 2a. Similarly, VO_2_ at AT showed a stronger negative correlation (r = −0.391, *p* = 0.009), depicted in Figure 2b. Additionally, peak VE correlated negatively with quantitative LV [^99m^Tc]-DPD uptake (r = −0.348, *p* = 0.021), as shown in Figure 2c. Further correlation results are provided in Table 2.

### 3.3. Correlation Between CPET, LV [^99m^Tc]-DPD Quantification and Key Clinical, Laboratory, and Imaging Parameters

Considering the multifaceted impact of transthyretin amyloidosis, significant correlations were observed between key CPET parameters, [^99m^Tc]-DPD retention index, and various clinical, laboratory, and imaging metrics. Notably, an advanced stage of HF showed a significant negative correlation with peak VO_2_ (r = −0.549, *p* < 0.001). Conversely, this parameter positively correlated with VE/VCO_2_ slope as well as [^99m^Tc]-DPD retention index (r = 0.324, *p* = 0.032). Expectedly, both CPET parameters correlated with the 6MWT distance (peak VO_2_: r = 0.384, *p* = 0.010, VE/VCO_2_ slope: r = −0.451, *p* < 0.001).

Markers of cardiac and renal function were also evaluated and showed significant correlations with CPET-derived variables. Higher NT-proBNP levels were strongly associated with lower peak VO_2_ (r = −0.530, *p* < 0.001) and higher VE/VCO_2_ slope (r = 0.348, *p* = 0.022). Troponin T, similarly, correlated negatively with peak VO_2_ (r = −0.431, *p* < 0.001) and positively with VE/VCO_2_ slope (r = 0.587, *p* < 0.001). eGFR displayed a positive correlation with peak VO_2_ (r = 0.402, *p* < 0.001) and negative with VE/VCO_2_ slope (r = −0.390, *p* < 0.001).

While most echocardiographic measures showed no significant correlation with CPET or quantitative [^99m^Tc]-DPD indices, right ventricle global longitudinal strain exhibited a negative correlation with peak VO_2_ (r = −0.350, *p* = 0.023). Interestingly, only the right ventricle ejection fraction and native T1 time showed a notable correlation with peak VO_2_ and VE/VCO_2_ slope (r = 0.374, *p* = 0.038 and r = 0.464, *p* < 0.001, respectively). Further information is provided in Table 3.

### 3.4. Association of Key CPET Variables and Quantitative LV [^99m^Tc]-DPD Uptake with Survival and HF Hospitalizations

**Figure A1:**
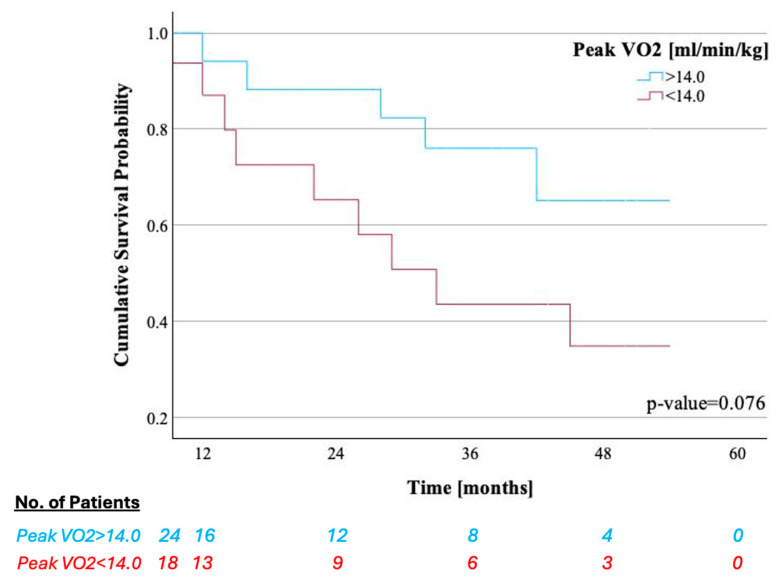
Kaplan–Meier estimates for the composite outcome (heart failure hospitalization or all-cause death) with peak oxygen uptake cut-off.

In the overall study cohort of 44 patients diagnosed with ATTR-CM, over a median follow-up period of 28.0 months (IQR: 8.0–43.0), there were 14 composite events of cardiovascular death or HF hospitalization. Among the patients (n = 19) with a peak VO_2_ below 14 mL/min/kg, nine patients (47%) reached the combined endpoint. In contrast, among those with a peak VO_2_ above 14, only 5 patients (20%) experienced the combined endpoint. Although the difference was not statistically significant, a positive trend toward event-free survival was observed for the patients with better baseline peak VO_2_ in Kaplan–Meier analysis [*p* = 0.076, HR 0.384 (95% Confidence interval (CI):0.128–1.151), *p* = 0.088], as illustrated in Figure A1.

**Figure A2:**
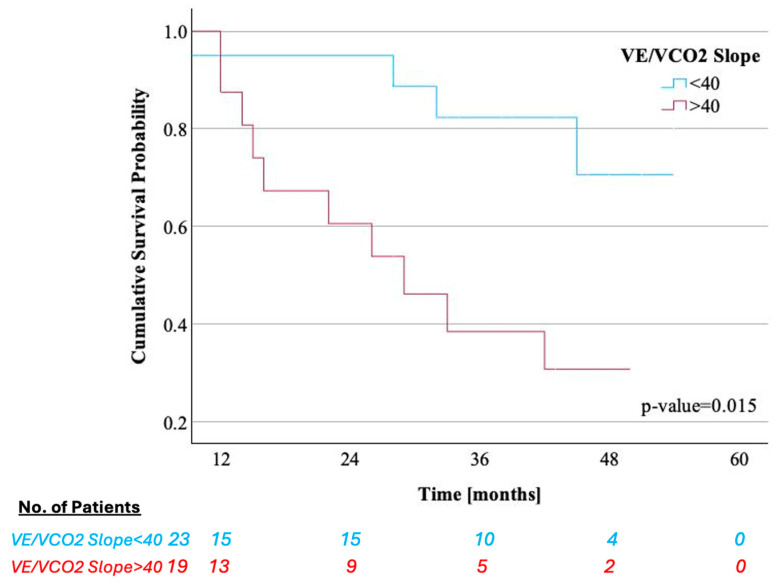
Kaplan–Meier estimates for the composite outcome (heart failure hospitalization or all-cause death) with VE/VCO_2_ slope cut-off.

The Kaplan–Meier survival curves illustrated clear survival discrepancies between the two groups defined by VE/VCO_2_ slope with a cut-off of 40. The group with a poorer VE/VCO_2_ slope (n = 20) demonstrated notably lower survival probability compared to the other group with a better VE/VCO_2_ slope, with 50% (n = 10) of patients reaching the combined endpoint [*p* = 0.015, HR 3.843 (95% CI:1.193–12.381), *p* = 0.024], as depicted in Figure A2.

**Figure A3:**
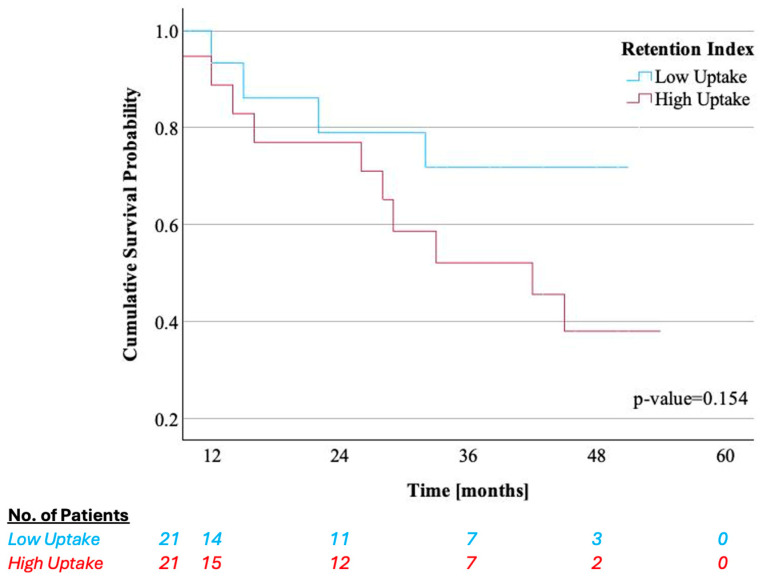
Kaplan–Meier estimates for the composite outcome (heart failure hospitalization or all-cause death) with DPD retention index.

Among the groups defined by [^99m^Tc]-DPD retention index, each consisting of 22 patients, the high [^99m^Tc]-DPD uptake cohort experienced a greater number of events [n = 10 (45.5%) vs. n = 4 (18%)]. The higher event rate in the high [^99m^Tc]-DPD group highlights a trend toward worse outcomes, though the difference was not statistically significant [*p*-value = 0.154, HR 2.274 (95% CI: 0.711–7.272), *p* = 0.166]. The Kaplan–Meier curves are represented in Figure A3.

Univariable Cox regression analysis for the combined endpoint demonstrated that following laboratory parameters: Troponin T (*p* = 0.017), eGFR (*p* = 0.007), along with CMR imaging native T1 time (*p* = 0.004), as well as CPET parameters such as peak VO_2_ (*p* = 0.019) and VO_2_ at anaerobic threshold (*p* = 0.016), were significant predictors of worse outcomes. After adjusting for these variables in a multivariable Cox regression model, only T1-Mapping remained as a significant predictor of adverse outcomes (*p* = 0.011). Detailed information can be found in Table A1.

## 4. Discussion

In this study, we investigated the relationship between functional capacity, as assessed by CPET, and quantitative LV [^99m^Tc]-DPD uptake in patients with ATTR-CM. Our analyses were focused on the interactions between these diagnostic and disease monitoring tools and their predictive capabilities for patient outcomes.

This is the first study that describes a significant inverse correlation between CPET-derived metrics, such as peak VO_2_, and [^99m^Tc]-DPD retention index, highlighting a functional impairment with rising myocardial amyloid burden. The second highlight of this study is a strong association between CPET metrics and essential clinical parameters, such as NYHA functional class and 6MWT. Furthermore, we demonstrated that higher quantitative cardiac [^99m^Tc]-DPD uptake and poorer CPET metrics were linked to a notably increased risk of adverse clinical outcomes.

The reduction in functional capacity due to amyloid burden is not merely a marker of disease severity but also a direct contributor to the symptomatic profile of ATTR-CM [21]. Patients with higher amyloid load, as defined by the greater LV [^99m^Tc]-DPD uptake, typically experience worse clinical outcomes and reduced quality of life due to diminished physical capacity [18,22]. Previous studies [23,24] have described that the amyloid proteins deposited in the extracellular matrix increase myocardial stiffness, which further directly impairs the ability of the heart to pump effectively and fill adequately during the cardiac cycle. These physiological changes significantly impact patients’ physical capacity, manifesting as reduced exercise tolerance that can be quantitatively measured through CPET metrics such as peak VO_2_ and VE/VCO_2_ slope [8,12]. By integrating both tools into patient monitoring, clinicians can assess the visible and functional amyloid burden more effectively. For example, a decline in CPET metrics alongside an increase in LV [^99m^Tc]-DPD uptake might indicate a progression of disease before clinical symptoms worsen, prompting earlier intervention. Conversely, improvements in CPET metrics could reflect a positive response to therapy, even before changes in LV [^99m^Tc]-DPD uptake are evident. Thus, our findings suggest that the combined use of CPET and quantitative [^99m^Tc]-DPD SPECT/CT, alongside clinical, laboratory, and imaging assessments, can provide a comprehensive assessment by correlating the extent of amyloid deposition with its functional impact.

To assess FC, we performed not only CPET as the gold standard, but also 6MWT. Despite similar 6MWT performance between groups, CPET parameters identified a remarkable difference between the two study groups, as shown in Table 1. While 6MWT is a submaximal test, it primarily reflects the patient’s ability to perform daily activities. It lacks the sensitivity to distinguish subtle differences in cardiopulmonary function between patients at varying stages of disease. In contrast, CPET is a maximal effort test that provides an integrated assessment of the cardiovascular, pulmonary, and muscular systems. Key CPET-derived metrics offer detailed physiological insight into both cardiac and peripheral muscle/metabolic limitations to exercise. This allows CPET to uncover impairments even when 6MWT shows preserved performance.

In our study, patients in the high LV [^99m^Tc]-DPD uptake cohort exhibited significantly elevated levels of NT-proBNP, reflecting increased myocardial wall stress and disease severity, and reduced eGFR compared to those in the low LV [^99m^Tc]-DPD uptake cohort (Table 1). Elevated NT-proBNP, a marker of cardiac stress and dysfunction, and decreased eGFR, indicative of renal impairment, are both reflective of the advanced disease stage, as previously introduced by Gillmore et al. [25]. In cardiac amyloidosis, amyloid deposition stiffens the ventricular walls, impairing diastolic filling and resulting in elevated intracardiac pressures. This hemodynamic burden, especially under conditions of volume overload, is a major stimulus for NT-proBNP synthesis and release. Our analysis also revealed that the parameters used in the National Amyloidosis Center staging system correlate significantly with CPET outcomes. Higher NT-proBNP and lower eGFR were associated with worse CPET metrics, such as reduced peak VO_2_ and higher VE/VCO_2_ slope (Table 3). These correlations are evident in our data and underscore the CPET’s ability to reflect broader exercise physiological disruptions caused by amyloidosis.

Further exacerbating the clinical scenario is the NYHA classification, where more patients in the high DPD uptake group were classified as NYHA Class III. The predominance of NYHA Class III in the high uptake group underscores the severity of heart failure in these patients and the substantial impact of amyloid deposition on cardiac function [26]. In our study, we observed a significant correlation between higher NYHA classes and both increased [^99m^Tc]-DPD uptake and poor CPET metrics. This correlation is pivotal because it links quantitative measures of myocardial amyloid burden and functional impairment directly with patient-reported symptom severity.

Consistent with findings from previous studies, our analysis confirmed the impairment of the right ventricle in patients with ATTR-CM [27,28]. This early dysfunction is particularly manifest in the reduced strain and ejection fraction of the right ventricle, which are sensitive indicators of early myocardial damage before overt clinical symptoms become apparent. The relationship between reduced peak VO_2_ and right ventricle dysfunction underscores the systemic nature of cardiac amyloidosis, where amyloid deposition not only affects myocardial stiffness and ventricular filling but also the contractile efficiency of the heart.

In ATTR-CM, understanding the relationship between disease progression and patient outcomes is crucial for effective management. In our study, we explored the implications of cardiac functional assessments and myocardial amyloid burden on the outcomes of patients with ATTR-CM. Many studies have demonstrated the useful prognostic utility of key CPET parameters in patients with cardiac amyloidosis [9,29,30]. Based on the established cut-off of 14 mL/min/kg for peak VO2 [9,31], we were able to confirm this in our study cohort. Patients with peak VO2 above this threshold generally exhibited better survival rates, underscoring peak VO2 as a crucial indicator of physical health and cardiac function in ATTR-CM (Figure A1).

Another important prognostic CPET marker, VE/VCO2 Slope, provides insight into ventilatory efficiency and has been shown in our study to significantly impact survival (Figure A2). Patients with VE/VCO2 Slope greater than 40 had markedly worse survival, which also corresponded with a few previous studies [9,32].

Recent findings by Pugliatti et al. have shown that CPET is a sensitive tool for detecting functional improvement following tafamidis therapy, even when echocardiographic parameters remain unchanged. This reinforces the value of CPET not only for baseline risk stratification but also for longitudinal monitoring of treatment response [33].

Although the prognostic value and accuracy of LV [^99m^Tc]-DPD quantification with SPECT/CT imaging have been established in several studies [18,34], we could show only a trend towards poorer survival in the high uptake cohort. Despite the lack of statistical significance, which may be attributed to the small sample size, this diagnostic tool remains crucial for patient risk stratification.

## 5. Limitations

This study, while providing valuable insights into the prognostic utility of CPET and DPD SPECT/CT imaging in ATTR-CM, has several limitations that must be mentioned. First, the data were collected from a single center, which may limit the generalizability of the findings. The patient population at a single institution may not fully represent the broader demographic and genetic diversity of ATTR-CM patients. Multicenter studies would be beneficial to validate our findings across diverse populations and healthcare settings. Secondly, the small sample size of our cohort limits the statistical power of this study, which may contribute to the lack of significant findings in some of the survival analyses. This limitation is particularly relevant when assessing the impact of high DPD uptake on survival, where the trend observed did not reach statistical significance. Another potential limitation of our study is the influence of polyneuropathy on CPET performance. Since peripheral neuropathy can impair muscle strength and coordination, it may affect the exercise capacity independently of cardiac function. However, in our cohort, the prevalence of polyneuropathy was comparable between the low and high DPD uptake groups, minimizing the risk of confounding in our comparative analysis.

## 6. Conclusions

Our findings suggest that the assessment of myocardial amyloid burden through LV [^99m^Tc]-DPD uptake quantification, coupled with regular CPET evaluations, should be considered integral to the management of ATTR-CM. Such an approach not only aids in diagnosing and staging the disease but also provides valuable prognostic information that can guide clinical decisions aimed at improving patient outcomes.

## Figures and Tables

**Table 1:** Baseline characteristics of our study cohort.

Variables	All (n = 44)	Low [^99m^Tc]-DPD Uptake (n = 22)	High [^99m^Tc]-DPD Uptake (n = 22)	*p*-Value
** Demographic and clinical parameters **				
Age, years	78.0 (73.0–78.0)	79.0 (72.0–80.0)	75.0 (71.0–79.0)	0.689
Sex, Male	36 (82.0)	20 (91.0)	16 (73.0)	0.240
Body Mass Index, kg/m^2^	27.0 (25.1–28.7)	24.5 (24.0–27.3)	28.0 (25.1–31.8)	0.124
NYHA Functional Class				**0.025**
Class I	6 (14.0)	5 (23.0)	1 (4.0)	
Class II	22 (50.0)	13 (59.0)	9 (41.0)	
Class III	16 (36.0)	4 (18.0)	12 (55.0)	
6 min Walk Distance, m	422.0 (366.1–458.3)	458.0 (347.0–496.1)	405.0 (341.2–462.1)	0.222
ATTRv	5 (11.0)	2 (9.0)	3 (14.0)	1.000
** Comorbidities **				
Arterial hypertension	24 (54.0)	10 (45.0)	14 (64.0)	0.364
Atrial fibrillation or flutter	26 (59.0)	13 (59.0)	13 (59.0)	1.000
Coronary artery disease	13 (29.0)	10 (45.0)	3 (14.0)	**0.045**
Carpal tunnel syndrome	25 (57.0)	13 (59.0)	12 (54.0)	1.000
Polyneuropathy	30 (68.0)	14 (64.0)	16 (73.0)	0.747
** Concomitant medication **				
Beta-blockers	18 (41.0)	9 (41.0)	9 (41.0)	1.000
MRA	23 (52.0)	9 (41.0)	14 (64.0)	0.227
Diuretics	30 (68.0)	13 (59.0)	17 (77.0)	0.332
Anticoagulants	26 (59.0)	13 (59.0)	13 (59.0)	1.000
Statins	21 (48.0)	11 (50.0)	10 (45.5)	1.000
** Laboratory parameters **				
NT-proBNP, pg/mL	1876.0 (1611.0–2595.3)	1364.0 (1051.0–2063.0)	2341.0 (1844.0–3506.4)	**0.029**
Troponin T, ng/L	41.0 (39.2–57.3)	39.0 (33.7–62.0)	46.0 (36.1–61.2)	0.916
eGFR, mL/min/1.73 m^2^	68.1 (59.1–75.4)	79.0 (64.0–85.0)	58.0 (47.0–72.2)	0.126
** Cardiopulmonary Exercise Testing **				
Peak VO_2_, mL/min/kg	15.0 (15.0–18.0)	16.1 (15.0–19.4)	12.7 (11.7–14.4)	**0.002**
VO_2_ at AT, mL/min/kg	10.0 (10.2–12.3)	11.4 (10.7–14.3)	9.2 (9.0–10.8)	**0.003**
Peak O_2_ Pulse, mL/beat	11.4 (10.4–12.4)	11.4 (10.2–13.6)	11.4 (9.3–12.3)	0.379
VE/VCO_2_ slope	39.4 (36.9–41.6)	39.3 (35.1–43.0)	39.7 (36.5–42.5)	0.606
Peak workload, Watt	80.0 (68.2–88.2)	90.0 (77.3–108.1)	56.0 (53.0–73.4)	**0.003**
Peak VE, L/min	52.5 (52.0–63.2)	60.0 (57.0–73.2)	45.0 (43.8–57.0)	**0.004**
** Nuclear imaging parameters **				
Perugini Grading Scale				0.760
2	18 (41.0)	8 (36.0)	10 (45.0)	
3	26 (59.0)	14 (64.0)	12 (54.0)	
[^99m^Tc]-DPD retention index	5.0 (4.3–6.0)	2.8 (2.4–3.3)	6.8 (6.5–8.0)	**<0.001**
** Transthoracic echocardiography parameters **				
Interventricular septum, mm	18.0 (17.4–20.2)	19.0 (17.1–21.3)	17.0 (16.4–20.5)	0.540
LV ejection fraction, %	51.0 (47.1–53.0)	52.0 (47.3–54.4)	50.0 (44.4–53.4)	0.518
LV end-diastolic diameter, mm	43.0 (42.0–45.5)	43.0 (40.5–46.0)	44.0 (41.0–47.2)	0.556
LV global longitudinal strain, -%	12.0 (13.5–11.4)	13.2 (15.0–11.5)	12.0 (13.0–10.4)	0.162
RV end-diastolic diameter, mm	34.0 (32.3–36.0)	34.0 (31.2–35.5)	34.0 (32.0–37.0)	0.787
RV global longitudinal strain, -%	15.0 (16.1–13.0)	16.3 (18.1–13.4)	12.0 (15.3–11.2)	0.166
** Cardiac magnetic resonance imaging parameters (n = 32) **				
Interventricular septum, mm	18.3 (16.4–21.1)	19.0 (15.2–21.0)	17.6 (15.1–24.4)	0.737
LV ejection fraction, %	47.1 (43.4–52.0)	46.0 (40.3–52.0)	49.0 (42.2–56.0)	0.766
RV ejection fraction, %	47.5 (42.0–51.0)	45.3 (42.0–53.0)	50.0 (36.4–54.0)	0.518
MOLLI-ECV, %	45.0 (41.0–51.0)	43.0 (35.3–51.0)	46.0 (42.1–56.0)	0.450
T1-Mapping, ms	1112.0 (1089.4–1158.3)	1113.0 (1069.1–1166.0)	1110.0 (1069.1–1166.0)	0.734

Continuous variables are presented as median and interquartile range (IQR), and categorical variables as numbers and percentages. Abbreviations: AT, anaerobic threshold; ATTRv, variant transthyretin amyloid; [^99m^Tc]-DPD, [^99m^Tc]-3,3-diphosphono-1,2-propanodicarboxylic acid; ECV, extracellular volume; eGFR, estimated glomerular filtration rate; LV, left ventricle; MRA, mineralocorticoid receptor antagonist; NT-proBNP, N-terminal prohormone of brain natriuretic peptide, NYHA, New York Heart Association functional class; RV, right ventricle; VE, minute ventilation; VE/VCO_2_ ventilation to cardon dioxide production; VO_2_, oxygen consumption.

**Table 2:** Correlations between CPET and LV [^99m^Tc]-DPD retention index.

CPET Variable	Correlation Coefficient	*p*-Value
Peak VO_2_, mL/min/kg	**−0.355**	**0.018**
VO_2_ at AT, mL/min/kg	**−0.391**	**0.009**
Peak O_2_ Pulse, mL/beat	0.075	0.629
VE/VCO_2_ slope	0.125	0.419
Peak workload, Watt	0.262	0.082
Peak VE, L/min	**−0.348**	**0.021**

Variables are presented as median and interquartile range (IQR). Abbreviations: LV, left ventricle; VE, minute ventilation; VE/VCO_2,_ ventilation to carbon dioxide production; VO_2_, oxygen consumption.

**Table 3:** Correlation between CPET, quantitative [^99m^Tc]-DPD LV uptake, and key clinical, laboratory, and imaging parameters.

Variables	Peak VO_2_, mL/min/kg	VE/VCO_2_ Slope	[^99m^Tc]-DPD Retention Index
** Clinical parameters **	Correlation coefficient	Correlation coefficient	Correlation coefficient
Body Mass Index, kg/m^2^	−0.197	−0.085	0.299 *
NYHA Functional Class ≥ III	−0.549 **	0.324 *	0.324 *
6-min Walk Distance, m	0.384 *	−0.451 **	0.012
** Laboratory parameters **			
NT-proBNP, pg/mL	−0.530 **	0.348 *	0.261
Troponin T, ng/L	−0.431 **	0.587 **	0.028
eGFR, mL/min/1.73 m^2^	0.402 **	−0.390 **	−0.138
** Transthoracic echocardiography parameters **			
Interventricular septum, mm	−0.041	0.312 *	0.119
LV ejection fraction, %	0.283	0.033	0.062
LV end-diastolic diameter, mm	−0.102	0.299 *	−0.001
LV global longitudinal strain, -%	−0.282	0.236	0.283
RV end-diastolic diameter, mm	−0.254	0.032	−0.031
RV global longitudinal strain, -%	−0.350 *	0.289	0.291
** Cardiac magnetic resonance imaging parameters **	n = 32	n = 17	n = 15
Interventricular septum, mm	−0.103	0.214	0.250
LV ejection fraction, %	0.211	−0.016	0.048
RV ejection fraction, %	0.374 *	−0.104	−0.003
MOLLI-ECV, %	−0.149	0.302	0.315
Native T1 time, ms	−0.276	0.464 **	0.231

Table 3 shows the simple correlation between key CPET, [^99m^Tc]-DPD retention index, and key clinical, laboratory, and imaging parameters. Correlations were assessed using the Spearman coefficient, which is reported in the table. * *p*-value < 0.05, ** *p*-value < 0.001. Abbreviations: [^99m^Tc]-DPD, [^99m^Tc]-3,3-diphosphono-1,2-propanodicarboxylic acid; ECV, extracellular volume; eGFR, estimated glomerular filtration rate; LV, left ventricle; NT-proBNP, N-terminal prohormone of brain natriuretic peptide, NYHA, New York Heart Association functional class; RV, right ventricle; VE, minute ventilation; VE/VCO_2_ ventilation to cardon dioxide production; VO_2_, oxygen consumption.

## Data Availability

Data will be shared upon reasonable request to the corresponding author.

## Abbreviations

The following abbreviations are used in this manuscript:

6MWT	6 min walk test
ATTR-CM	Transthyretin amyloid cardiomyopathy
AT	Anaerobic threshold
BMI	Body mass index
CMR	Cardiac magnetic resonance
CPET	Cardiopulmonary exercise testing
[^99m^Tc]-DPD	[^99m^Tc]-3,3-diphosphono-1,2-propanodicarboxylic acid
ECV	Extracellular volume
FC	Functional capacity
HF	Heart failure
LV	Left ventricle
NYHA	New York Heart Association
[^99m^Tc]-PYP	[^99m^Tc]-pyrophosphate
SPECT/CT	Single photon emission computed tomography/computed tomography
TTE	Transthoracic echocardiography
VCO_2_	Carbon dioxide production
VE	Pulmonary ventilation
VE/VCO_2_ slope	Ventilation to carbon dioxide production
VO_2_	Oxygen consumptions

## Appendix A

**Table A1:** Cox regression analysis for the composite endpoint.

Variables	Hazard Ratio (95% CI)	*p*-Value	Hazard Ratio (95% CI)	*p*-Value
	Univariable Regression		Multivariable Regression	
** Demographic and clinical parameters **				
Age, years	0.965 (0.908–1.026)	0.252		
Sex, Male	0.553 (0.173–1.768)	0.318		
Body Mass Index, kg/m^2^	1.015 (0.923–1.116)	0.762		
NYHA Functional Class ≥ III	2.216 (0.740–6.636)	H00.155		
6-min Walk Distance, m	0.999 (0.995–1.003)	0.694		
** Concomitant medication **				
Beta-blockers	0.947 (0.316–2.842)	0.923		
MRA	2.849 (0.791–10.262)	0.109		
Diuretics	3.404 (0.444–26.077)	0.238		
** Laboratory parameters **				
NT-proBNP, pg/mL	1.000 (1.000–1.000)	0.422		
Troponin T, ng/L	1.022 (1.004–1.041)	**0.017**	1.026 (0.985–1.069)	0.213
eGFR, mL/min/1.73 m^2^	0.967 (0.944–0.991)	**0.007**	0.985 (0.927–1.045)	0.612
** Cardiopulmonary Exercise Testing **				
Peak VO_2_, mL/min/kg	0.831 (0.712–0.970)	**0.019**	0.681 (0.327–1.415)	0.303
VO_2_ at AT, mL/min/kg	0.593 (0.387–0.908)	**0.016**	1.016 (0.431–2.397)	0.970
Peak O_2_ Pulse, mL/beat	0.860 (0.726–1.018)	0.079		
VE/VCO_2_ slope	1.040 (0.979–1.104)	0.206		
Peak workload, Watt	0.988 (0.968–1.008)	0.230		
Peak VE, L/min	0.981 (0.951–1.012)	0.234		
** Nuclear imaging parameters **				
Perugini Grading Scale	2.287 (0.637–8.210)	0.205		
DPD retention index	1.164 (0.951–1.423)	0.141		
** Transthoracic echocardiography parameters **				
Interventricular septum, mm	0.985 (0.874–1.110)	0.802		
LV ejection fraction, %	0.992 (0.939–1.048)	0.776		
LV end-diastolic diameter, mm	1.013 (0.932–1.101)	0.760		
LV global longitudinal strain, -%	1.058 (0.919–1.218)	0.430		
RV end-diastolic diameter, mm	1.016 (0.924–1.116)	0.750		
RV global longitudinal strain, -%	1.048 (0.947–1.159)	0.366		
** Cardiac magnetic resonance imaging parameters **	n = 32	n = 17	n = 15	
Interventricular septum, mm	1.039 (0.943–1.146)	0.438		
LV ejection fraction, %	1.013 (0.956–1.074)	0.653		
RV ejection fraction, %	1.001 (0.947–1.057)	0.979		
MOLLI-ECV, %	1.043 (0.975–1.115)	0.218		
Native T1 time, ms	1.011 (1.004–1.019)	**0.004**	1.017 (1.004–1.030)	**0.011**

Abbreviations: AT, anaerobic threshold; CI, confidence interval; DPD, [^99m^Tc]-3,3-diphosphono-1,2-propanodicarboxylic acid; ECV, extracellular volume; eGFR, estimated glomerular filtration rate; LV, left ventricle; MRA, mineralocorticoid receptor antagonist; NT-proBNP, N-terminal prohormone of brain natriuretic peptide, NYHA, New York Heart Association functional class; RV, right ventricle; VE, minute ventilation; VE/VCO_2_ ventilation to cardon dioxide production; VO_2_, oxygen consumption.
